# A New Guanidine-Core Small-Molecule Compound as a Potential Antimicrobial Agent against Resistant Bacterial Strains

**DOI:** 10.3390/antibiotics13070609

**Published:** 2024-06-29

**Authors:** Noelia Morata-Moreno, Ramón Pérez-Tanoira, Almudena del Campo-Balguerias, Fernando Carrillo-Hermosilla, Marcos Hernando-Gozalo, Carlos Rescalvo-Casas, Ana V. Ocana, Pedro Segui, Carlos Alonso-Moreno, Francisco C. Pérez-Martínez, Milagros Molina-Alarcón

**Affiliations:** 1 Department of Otorrinolaringology, Complejo Hospitalario Universitario, 02006 Albacete, Spain; noeliamoratamoreno95@hotmail.com (N.M.-M.); pedroto80@gmail.com (P.S.); 2 Departamento de Microbiología Clínica, Hospital Universitario Príncipe de Asturias, 28805 Madrid, Spain; ramontanoira@hotmail.com (R.P.-T.); marcos.torrejon1998@gmail.com (M.H.-G.); carlos.rescalvo@uah.es (C.R.-C.); 3 Departamento de Biomedicina y Biotecnología, Facultad de Medicina, Universidad de Alcalá, 28805 Madrid, Spain; 4 Unidad nanoDrug, Centro Regional de Investigaciones Biomédicas, Universidad de Castilla-La Mancha, 02008 Albacete, Spain; almudena.delcampo@uclm.es; 5 Departamento Química Inorgánica, Orgánica y Bioquímica, Facultad de Farmacia de Albacete-Centro de Innovación en Química Avanzada (ORFEO-CINQA), Universidad de Castilla-La Mancha, 02008 Albacete, Spain; 6 Departamento de Química Inorgánica, Orgánica y Bioquímica-Centro de Innovación en Química Avanzada (ORFEO-CINQA), Universidad de Castilla-La Mancha, 13071 Ciudad Real, Spain; fernando.carrillo@uclm.es; 7 Departamento de Química Orgánica y Química Inorgánica, Facultad de Farmacia, Universidad de Alcalá, 28805 Madrid, Spain; 8 Instituto de Investigación en Discapacidades Neurológicas (IDINE), University of Castilla-La Mancha, 02001 Albacete, Spain; ocanaav@gmail.com (A.V.O.); milagros.molina@uclm.es (M.M.-A.); 9 Department of Nursing, University of Castilla-La Mancha, 02071 Albacete, Spain

**Keywords:** *Staphylococcus aureus*, guanidine, antibacterial effect, *Escherichia coli*, *Pseudomonas aeruginosa*

## Abstract

The guanidine core has been one of the most studied functional groups in medicinal chemistry, and guanylation reactions are powerful tools for synthesizing this kind of compound. In this study, a series of five guanidine-core small molecules were obtained through guanylation reactions. These compounds were then evaluated against three different strains of *Escherichia coli*, one collection strain from the American Type Culture Collection (ATCC) of *E. coli* ATCC 35218, and two clinical extended-spectrum beta-lactamase (ESBL)-producing *E. coli* isolates (ESBL1 and ESBL2). Moreover, three different strains of *Pseudomonas aeruginosa* were studied, one collection strain of *P. aeruginosa* ATCC 27853, and two clinical multidrug-resistant isolates (PA24 and PA35). Among Gram-positive strains, three different strains of *Staphylococcus aureus*, one collection strain of *S. aureus* ATCC 29213, and two clinical methicillin-resistant *S. aureus* (MRSA1 and MRSA2) were evaluated. Minimum inhibitory concentration (MIC) and minimum bactericidal concentration (MBC) experiments were reported, and the drop plate (DP) method was used to determine the number of viable suspended bacteria in a known beaker volume. The results from this assessment suggest that guanidine-core small molecules hold promise as therapeutic alternatives for treating infections caused by clinical Gram-negative and Gram-positive bacteria, highlighting the need for further studies to explore their potential. The results from this assessment suggest that the chemical structure of CAPP4 might serve as the basis for designing more active guanidine-based antimicrobial compounds, highlighting the need for further studies to explore their potential.

## 1. Introduction

Antimicrobial resistance (AMR) remains a major public health concern worldwide [1]. AMR by pathogenic bacteria severely affects patients who are in a state of immunocompetence due to conditions or diseases such as cystic fibrosis, bronchiectasis, neutropenia, cancer, acquired immune deficiency syndrome, organ transplantation, intensive care unit admissions, uncontrolled diabetes mellitus, and burns, among others [2].

According to the European Center for Disease Prevention and Control (ECDC), more than 670,000 infections were reported due to bacteria resistant to antibiotics and approximately 33,000 people die from complications every year in Europe [3]. The effectiveness of well-established antibiotics has been increasingly compromised as the prevalence of multidrug resistant strains continues to rise globally each year [4]. This escalating issue is exacerbated by the inappropriate use of antibiotics, which contributes to the development of resistance mechanisms in bacterial strains such as *Escherichia coli*, *Pseudomonas aeruginosa*, and *Staphylococcus aureus* [5]. Therefore, there is an urgent need for novel compounds with antimicrobial properties effective against multidrug-resistant strains.

Testing compounds in clinical strains is a crucial step in the drug discovery and development process [6]. This approach helps ensure that potential therapies are effective, safe, and relevant to the treatment of human diseases. Additionally, it plays a significant role in addressing issues of drug resistance and advancing personalized medicine approaches [1].

In pharmaceutical chemistry, guanidines stand out as exceptionally versatile and intriguing organic molecules. They have been the subject of extensive studies over the years in the field of medicinal chemistry due to their wide-ranging applications. Classified as organosuperbases, guanidines have the capacity to accept protons in biological environments, facilitating stable hydrogen bonding with multiple biological species. Therefore, the guanidine core is present in the structure of numerous drugs for a wide range of therapeutic purposes. Drugs such as rosuvastatin, a hypocholesterolemic drug to prevent cardiovascular disease [7]; metformin, an antidiabetic drug [8]; guanabenz, an antihypertensive drug [9]; or methotrexate, an antineoplastic and immunomodulating agent [10], are some representative examples of guanidine-core compounds. In addition, drugs such as the antibiotic streptomycin, the antiviral drug type against influenza zanamivir, or the antimalarial drug proguanil, and the recently reported candidate for malaria treatment are additional examples of drugs employed as antimicrobials. These examples underscore the essential role of the guanidine core in the chemical structure of antimicrobial agents and highlight its significance in therapeutic drug development [11].

Guanidine synthesis relies on chemical transformations of electrophilic costly reagents, such as isothioureas, carbodiimides, or cyanamides, that often occur in low yields and require the use of toxic reagents, with the formation of undesirable by-products [12]. In the search for more efficient methods, guanylation reactions of amines with carbodiimides bring up an efficient and atom-economical catalytic methodology to be explored [13]. In particular, the use of commercially available compounds such as ZnEt_2_ as catalysts, and carbodiimides and amines as raw materials, might ease extending the scope of guanidine-core small molecules [14].

In this study, we conducted a screening of small-molecule compounds containing a guanidine core to identify novel drugs for the treatment of infectious diseases caused by Gram-negative and Gram-positive clinical strains. Guanidines were obtained by guanylation reactions, and minimum inhibitory concentration (MIC) and minimum bactericidal concentration (MBC) experiments as well as the drop plate (DP) method were carried out for assessment. Our findings allowed the identification of a novel guanidine-core candidate for treating infections caused by clinical strains, demonstrating the potential of the guanylation reaction approach in drug discovery.

## 2. Materials and Methods

### 2.1. General Procedure

To achieve a protective atmosphere, the reactions were carried out using standard Schlenk and glove-box techniques. The solvents were previously degassed and distilled using appropriate drying agents. CDCl_3_ was stored over activated 4 Å molecular sieves and degassed by several freeze–thaw cycles. By using a Varian FT-400 spectrometer (VARIAN Inc., Palo Alto City, CA, USA), all the nuclear magnetic resonance (NMR) experiments were performed in the deuterated solvents at 297 K. The equipment was provided with a 4 nucleus ASW PFG ^1^H/^19^F/^13^C/{^15^N-^31^P}. The ^1^H π/2 pulse length was adjusted for each sample. The ^1^H- and ^13^C{^1^H}-NMR chemical shifts (δ) are given in ppm relative to tetramethylsilane (TMS). Coupling constants (J) are documented in Hertz (Hz). The solvent signals were used as references and chemical shifts were converted to the TMS scale.

### 2.2. Synthesis of Guanidine Derivatives ***1**–**5***

The compounds **1**–**5** were obtained following procedures previously reported [14]. Briefly, 2.00 mmol of the amine was dissolved in THF under continuous stirring. To this solution, 0.03 mmol of ZnEt_2_ (1 M hexanes) was added. After 1 h of reaction, 2 mmol of N,N’-dicyclohexylcarbodiimide or N,N’-isopropylcarbodiimide in each case was added to the reaction mixture and left at 50 °C for 2 h. Finally, the remaining solvent was removed under a vacuum and the product was washed with hexane. Compounds **1**–**5** were obtained in very high yields as powered yellowish solids [13] (see ^1^H-NMR spectra of **1**–**5** in Figures S1–S5 of the Supporting Information to support the synthesis of the already reported guanidines).

### 2.3. Microbiological Studies

#### 2.3.1. Bacterial Strains

The reference strains from the American Type Culture Collection (ATCC) methicillin-susceptible *Staphylococcus aureus* ATCC 29213 and *Pseudomonas aeruginosa* ATCC 27853, and the clinical strains of methicillin-resistant *Staphylococcus aureus* (MRSA 1 and MRSA 2) and multidrug-resistant *Pseudomonas aeruginosa* (PA 24 and PA 35), used in these previous studies [15,16], were used. In addition, the laboratory strain *Escherichia coli* ATCC 35218 and clinical strains of extended-spectrum beta-lactamase-producing *Escherichia coli* (ESBL 1 and ESBL 2), isolated from patients diagnosed by the Clinical Microbiology Service of University Hospital Príncipe de Asturias (Alcalá de Henares, Spain), were included. Specifically, ESBL 1 was isolated from the bacteremia of a 75-year-old man and ESBL 2 from the urinary tract infection of an 87-year-old woman. All strains were kept frozen at −80 °C until the experiments were performed. Subsequently, they were thawed, seeded on colistin-nalidixic acid (CNA) agar and McConkey agar, if the bacterium was Gram-positive or negative, respectively, and incubated for 24 h at 37 °C and 5% CO_2_.

#### 2.3.2. MICs and MBCs

To analyze the antimicrobial properties of the **1**–**5** compounds, MIC and MBC studies were carried out against the planktonic forms of the bacteria mentioned above. The MIC is the concentration of an antimicrobial necessary to inhibit the growth of a finite microbial population and was determined according to Clinical Laboratory Standard Institute (CLSI) guidelines [17]. For this purpose, a bacterial inoculum with a bacterial concentration of 1 × 10^8^ colony-forming units per milliliter (CFU/mL) was prepared in 0.9% saline and diluted 1:100 in a Müller–Hinton broth (MHB). In a rounded-bottom 96-well plate (Thermo Fisher Scientific, Roskilde, Denmark), 100 µL of serial dilutions of the compounds in a concentration range of 256–0.25 mg/L and 100 µL of the diluted bacterial inoculum were added to each well. Subsequently, the plate was incubated for 24 h at 37 °C and 5% CO_2_. The MIC was evaluated by measuring the absorbance at 600 nm in a microtiter plate reader (Epoch ^TM^, BioTek Instruments, Winooski, VT, USA). This in turn was accompanied by serial dilutions of the compounds without microorganisms as negative absorbance controls.

For its part, the MBC is the concentration of an antimicrobial required to kill a finite bacterial population and was established according to the flash microbicidal method [18]. For this purpose, 180 µL of tryptose-soy broth (TSB) and 20 µL of the previous MIC plate were mixed in each well of a flat-bottomed plate. This plate was incubated for 24 h at 37 °C and 5% CO_2_. Finally, the MBC was determined by measuring the absorbance at 600 nm.

#### 2.3.3. Drop Plate Assays (DP)

For DP assays, 100 µL were taken from the positive control and MIC wells, and they were serially diluted with saline. CFU/mL was estimated using the drop plate (DP) method [19] on CNA agar plates for *S. aureus* and McConckey agar for *P. aeruginosa* and *E. coli*. The plates were incubated at 37 °C and 5% CO_2_ for 24 h.

### 2.4. Statistical Analysis

All concentration data were expressed as medians and interquartile ranges (IQRs). Calculations were performed with Microsoft Excell 2016 and statistical analysis with SPSS v20.0 (IBM Corp., Armonk, NY, USA). Data were assessed using a Student’s *t*-test and those with *p*-value ≤ 0.05 were established as statistically significant.

## 3. Results

### 3.1. Synthesis of Guanidine-Core-Bearing Small Molecules

**Scheme 1:**
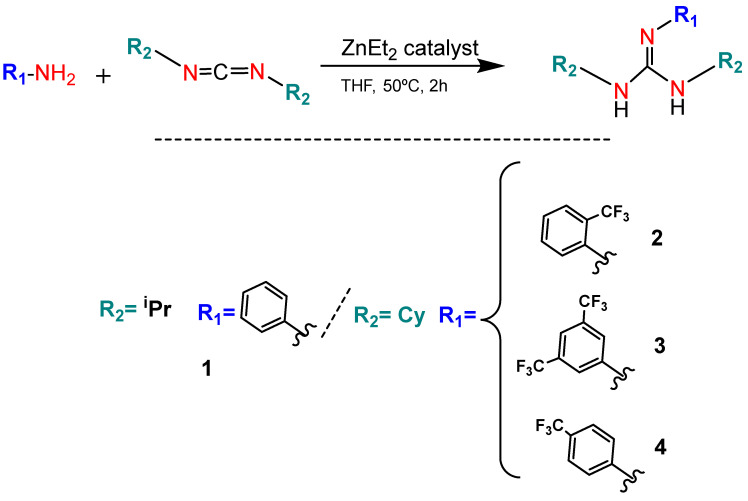
Synthesis procedure of compounds **1**–**5**.

Compounds **1**–**5** were obtained by guanylation reactions using the commercially available ZnEt_2_ as the catalyst (Scheme 1). Due to the formation of a zinc alkyl-amido species, subsequent nucleophilic addition to the carbodiimide became feasible; followed by amine protonolysis of intermediate guanidine species [14]. For the first screening, mono-substituted phenyl-guanidines including fluorinated functional groups (-CF_3_ in different positions) were proposed. To confirm the synthesis of the previously reported guanidines **1**–**5**, ^1^HNMR spectroscopy was conducted. The ^1^H-NMR structural elucidation is depicted in Figures S1–S5 of the Supporting Information.

### 3.2. Microbiological Studies

#### 3.2.1. MIC and MBC Studies

To determine the antibacterial capabilities of these compounds against planktonic cells, MIC and MBC studies were performed. Concerning *S. aureus* strains (Table 1), only the four guanidine showed in vitro good antibacterial activity, with the remaining four molecules completely lacking antimicrobial properties. For the *P. aeruginosa* strains (Table 2), the five molecules were unable to inhibit planktonic growth in vitro, showing very high MICs and MBCs (>256 mg/L) in all cases. Finally, compounds **1**–**5** did not show in vitro antimicrobial activity against the three *E. coli* strains used (Table 3), showing very high MICs and MBCs (>256 mg/L) in all cases.

#### 3.2.2. DP Assays

**Figure 1:**
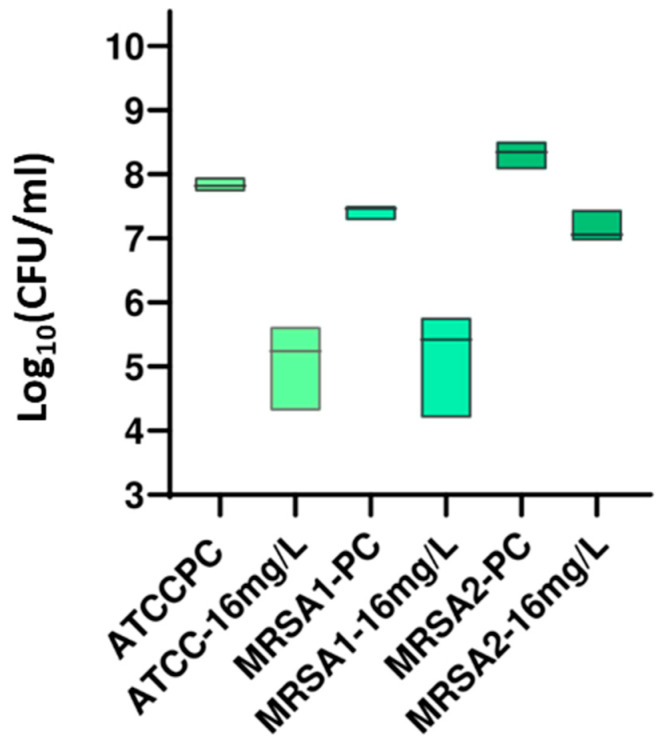
The inhibitory effect of compound **4** on the planktonic growth of strains of *Staphylococcus aureus* using the drop plate method. PC: Positive control; MRSA: Methicillin-resistant *Staphylococcus aureus*; ATCC: American Type Culture Collection; CFU: unit for the forming colony.

Based on the above results, the bacteriostatic effect of those compounds with the best antimicrobial activity (>20% inhibition) was determined by the DP method. Accordingly, this technique was applied for compound **4** in all bacterial strains, and for compound **3** only in *P. aeruginosa* strains. For the *S. aureus* strains, it was evidenced that a MIC of four caused an inhibition of bacterial growth of 99.71%, 98.90%, and 92.69% for the strains ATCC 29213, MRSA1, and MRSA2, respectively, with statistically significant differences for the first two isolates (*p*-value = 0.022 and *p*-value = 0.023, respectively) (Figure 1).

**Figure 2:**
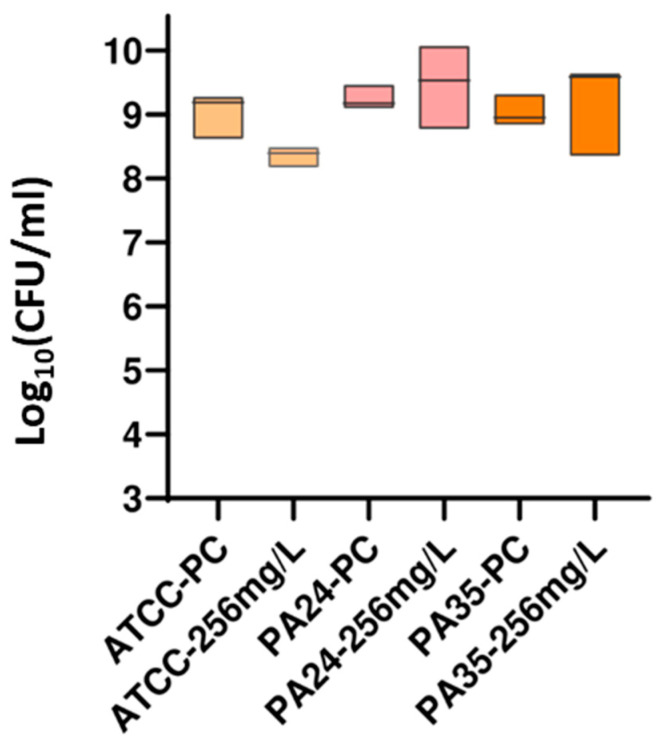
The inhibitory effect of compound **4** on the planktonic growth of strains of *Pseudomonas aeruginosa* using the drop plate method. PC: Positive control; ATCC: American Type Culture Collection; CFU: colony-forming unit.

**Figure 3:**
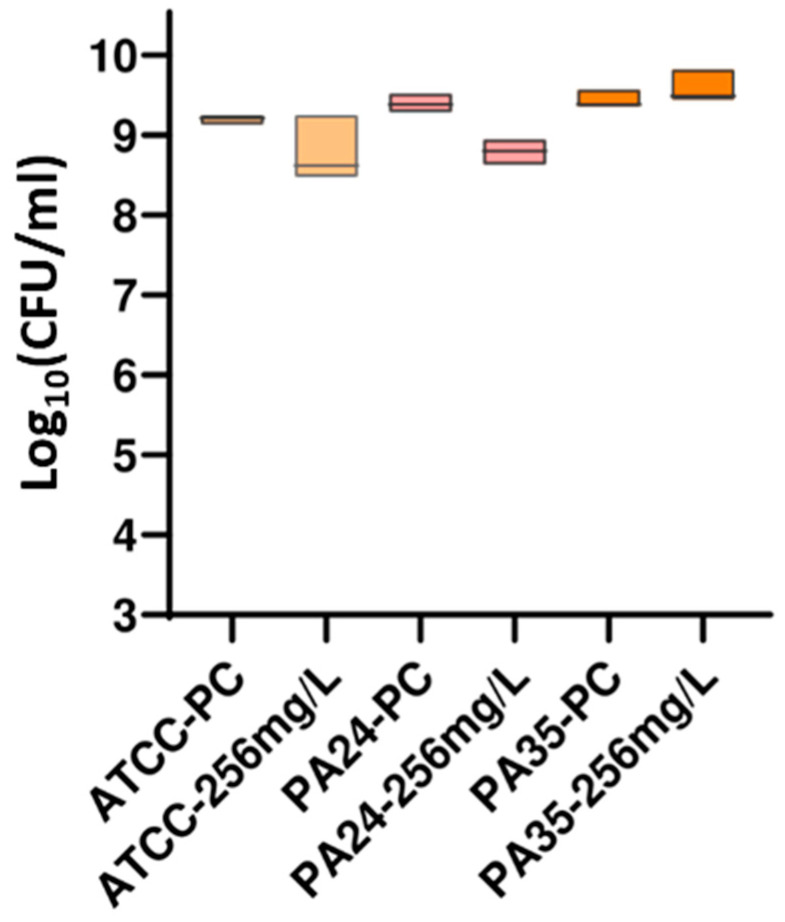
The inhibitory effect of compound **3** on the planktonic growth of strains of *Pseudomonas aeruginosa* using the drop plate method. PC: Positive control; ATCC: American Type Culture Collection; CFU: colony-forming unit.

Compound **4** at a concentration of 256 mg/L was able to decrease in vitro the planktonic growth of the *P. aeruginosa* ATCC 27853 strain by 81.58% without statistically significant differences (*p*-value = 0.165) (Figure 2). On the other hand, compound **3** only decreased microbial growth concerning a control by 47.92% and 74.60% for the strains ATCC 27853 and PA24, respectively (Figure 3). However, these inhibitions were not statistically significant (*p*-value = 0.329 and *p*-value = 0.056, respectively).

**Figure 4:**
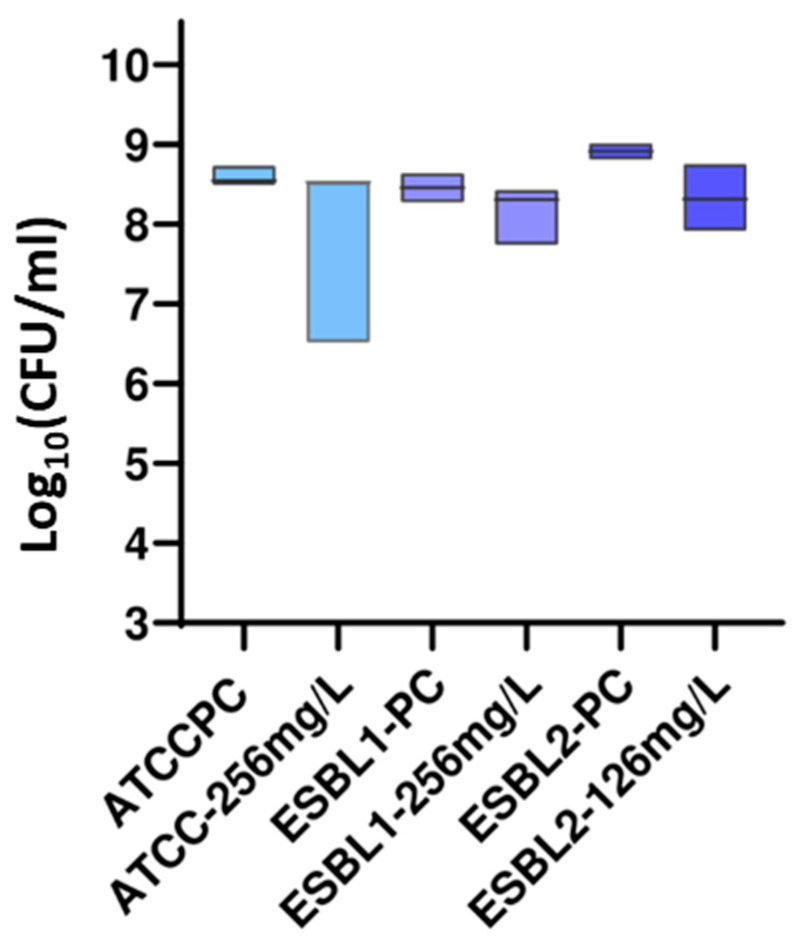
The inhibitory effect of compound **4** on the planktonic growth of strains of *Escherichia coli* using the drop plate method. * Significance *p* < 0.05. PC: Positive control; ESBL: extended-spectrum beta-lactamase; ATCC: American Type Culture Collection; CFU: colony-forming unit.

Regarding the *E. coli* strains, compound **4** at the concentration of 256 mg/L showed some antimicrobial characteristics, as it was able to reduce in vitro the bacterial growth by 43.36%, 35.85%, and 75.58% for the strains ATCC 35218, ESBL1, and ESBL2, respectively. However, statistically significant differences were only found for the ESBL2 strain (*p*-value = 0.008) (Figure 4).

## 4. Discussion

It is a known fact that time-consuming and expensive screening is needed to pick out promising candidates from the vast number of compounds that can be synthesized. Thus, novel techniques to make this screening quicker, more efficient, and less expensive are constantly being developed. Thus, candidates must be synthesized in the first instance, and synthetic methodologies need to be straightforward and atom-economical as a way of improving screening [20,21]. Guanylation reactions using commercially available catalysts meet this requirement. Such catalytic reactions allow fast and efficient generation of guanidine-core small molecules [14].

Guanidine structures have been the cornerstone of many pharmaceuticals [22]. The devotion to such structures allowed drug designers to find successful therapies to prevent cardiovascular diseases and treat diabetes, hypertense, peptic ulcers, and even rheumatoid arthritis. Examples also include the antibiotics streptomycin, trimethoprim, and chlorhexidine as well as the antimalarial medication proguanil [11]. The presence of guanidine moieties has proven effective in augmenting the antimicrobial activity of various compounds. In the case of antibacterial activity, guanidine-containing compounds have shown efficacy against Gram-positive and Gram-negative bacterial strains as well as modulators of drug resistance [11]. In this regard, Dantas et al., in 2018, reported a new family of antibacterial compounds with an aminoguanidine functional group as possible inhibitors of the NorA efflux pump in *S. aureus* [23]. More recently, Deng and Song, in 2020, reported on a different sequence of alkynyl compounds linked to aminoguanidine that showed significant antibacterial effects against both Gram-positive and Gram-negative bacteria [24]. In addition, several guanidinomethyl biaryl compounds were described as having antibacterial activities, showing that these compounds bind the bacterial cell division protein FtsZ and promote FtsZ self-polymerization [25].

Given the interest in guanidines as biologically relevant moieties in drug design and the recent advances made over the last decade, it appears clear that guanidine-containing compounds hold substantial promise for advancing antimicrobial drug discovery and development.

The classical synthesis of guanidines is based on approaches that require toxic, poorly available, and costly reagents to give rise to low yields for a comprehensive array of substrates and produce undesirable substances. In this regard, guanylation reactions might bring up efficient alternatives for the synthesis of these systems. In this study, phenyl-guanidine derivatives were obtained in excellent yields using a catalytic guanylation reaction with 100% atom economy in a virtually waste-free process from relatively cheap and widely available starting materials. The design of this first screening of guanidine-core small-molecule compounds for treating infections caused by clinical Gram-negative and Gram-positive strains relies on the incorporation of fluorinated functional groups into the molecular structure. Such a fluorinated functional group is a key medicinal chemistry bioisostere used to alter the bioavailability, metabolic stability, affinity, and lipophilicity of novel pharmaceuticals, as an alternative to chlorine or methyl groups [26]. Therefore, five guanidines were proposed: two of them without any -CF_3_ groups within the structure (compounds **1** and **2**), as a control of our hypothesis on the impact of the presence of these groups on the activity of the compounds studied, and three of them with the incorporation of -CF_3_ moieties in different positions of the aromatic ring (compounds **3**–**5**).

Once the compounds were obtained, the screening started with their assessment of clinical isolates Gram-positive and Gram-negative strains. To determine the antibacterial properties of these compounds, *Staphylococcus aureus* was chosen as a model for Gram-positive bacteria and *Escherichia coli* and *Pseudomonas aeruginosa* as models for Gram-negative bacteria. Collection strains have a lower genetic load than clinical strains isolated from patients because they are laboratory-adapted strains, which lose genes due to several passages on the culture medium. It is worth noting that the importance of test compounds in clinical strains is a critical aspect of drug discovery and development, because of the need to ensure that potential therapies are effective, safe, and relevant to the treatment of human diseases.

In this study, the antibacterial effects of the guanidine-based agents against three *E. coli* strains (ATCC 35218, ESBL1, and ESBL2), three *P. aeruginosa* strains (ATCC 27853, PA24, and PA35), and three *S. aureus* strains (ATCC 29213, MRSA1, and MRSA2) were carried out. The results supported higher effects against Gram-positive clinical isolates, maybe due to the fact that guanidine groups are positively charged at a physiological pH, being more likely to enter bacteria through porous channels than uncharged compounds. Moreover, out of these five compounds, compound **4** showed the highest antibacterial activity against clinical Gram-negative and Gram-positive strains. A recent study using guanidine compounds showed similar results against a panel of bacteria, including pertinent clinical isolates [27]. Thus, linear alkyl- and macrocyclic compounds containing several guanidine groups within the macrostructure showed a synthetic broad-spectrum antibacterial agent with MIC values of 0.5–1 mg/L against *E. coli*, *Enterococcus faecalis*, *Klebsiella pneumoniae*, and *Staphylococcus epidermidis*. In our study, in which we explored exclusively small molecules containing one guanidine group, the incorporation of two -CF_3_ groups in the compound **4** derivative increased effects against planktonic Gram-positive strains, although slight effects were also observed against Gram-negative ones. Based on our findings, it can be hypothesized that the pattern of substitutions surrounding the guanidine moiety has a significant impact on activity, which may be explained in terms of better bioavailability based on a higher lipophilic character. In this regard, clinical drugs such as the antitubercular streptomycin and the trimethoprim used for the treatment of urinary tract infections are examples of anti-infective agents containing a guanidine group which points to its relevance as a biologically interesting moiety in identifying and developing new antimicrobial agents [28].

The discovery of compound **4** as an agent for treating infections might lead to improved synthetic routes to discover new derivatives able to act against bacterial strains at non-toxic doses for eucaryotic cells. Thus, further studies to understand the main mechanism of action of these structures and to demonstrate their in vivo efficacy are ongoing in our laboratories.

## 5. Conclusions

In conclusion, the chemical structure of compound **4** might serve as the basis for the design of more active guanidine-based antimicrobial compounds and pave the way for the clinical development of these compounds as alternatives for the treatment of infections. The simply accessible synthesis of such compounds by guanylation reactions makes compound **4** a good starting point to work with to improve the pharmacological profile of phenyl-guanidine derivatives and, therefore, for further evaluation in vivo of guanidines as potential chemotherapeutic agents.

## Figures and Tables

**Table 1:** Antimicrobial properties of compounds **1**–**5** against *Staphylococcus aureus* strains.

Strain	Compound	MIC ^a^ (mg/L)	MBC ^b^ (mg/L)	%Inhibition at MIC Wells ^c^
ATCC 29213	**1**	>256	>256	19.24
	**2**	>256	>256	12.58
	**3**	>256	>256	19.45
	**4**	16	128	99.53
	**5**	>256	>256	17.33
MRSA 1	**1**	>256	>256	18.26
	**2**	>256	>256	11.40
	**3**	>256	>256	18.12
	**4**	16	32	100.00
	**5**	>256	>256	26.24
MRSA 2	**1**	>256	>256	15.70
	**2**	>256	>256	23.25
	**3**	>256	>256	24.13
	**4**	16	128	100.00
	**5**	>256	>256	15.84

^a^ MIC: Minimum inhibitory concentration (mg/L); ^b^ MBC: minimum bactericidal concentration (mg/L); ^c^ by measuring the absorbance at 600 nm.

**Table 2:** Antimicrobial properties of compounds **1**–**5** against *Pseudomonas aeruginosa* strains.

Strain	Compound	MIC ^a^ (mg/L)	MBC ^b^ (mg/L)	%Inhibition at MIC Wells ^c^
ATCC 27853	**1**	>256	>256	14.24
	**2**	>256	>256	13.08
	**3**	>256	>256	21.09
	**4**	>256	>256	19.04
	**5**	>256	>256	15.82
PA 24	**1**	>256	>256	28.10
	**2**	>256	>256	4.11
	**3**	>256	>256	33.75
	**4**	>256	>256	22.20
	**5**	>256	>256	14.88
PA 35	**1**	>256	>256	38.52
	**2**	>256	>256	3.36
	**3**	>256	>256	41.83
	**4**	>256	>256	21.54
	**5**	>256	>256	3.72

^a^ MIC: Minimum inhibitory concentration (mg/L); ^b^ MBC: minimum bactericidal concentration (mg/L); ^c^ by measuring the absorbance at 600 nm.

**Table 3:** Antimicrobial properties of compounds **1**–**5** against *Escherichia coli* strains.

Strain	Compound	MIC ^a^ (mg/L)	MBC ^b^ (mg/L)	%Inhibition at MIC Wells ^c^
ATCC 35218	**1**	>256	>256	0
	**2**	>256	>256	0
	**3**	>256	>256	0
	**4**	>256	>256	0
	**5**	>256	>256	0
ESBL 1	**1**	>256	>256	0
	**2**	>256	>256	5.49
	**3**	>256	>256	12.36
	**4**	>256	>256	28.54
	**5**	>256	>256	0
ESBL 2	**1**	>256	>256	0
	**2**	>256	>256	0
	**3**	>256	>256	5.33
	**4**	>256	>256	40.61
	**5**	>256	>256	0

^a^ MIC: Minimum inhibitory concentration (mg/L); ^b^ MBC: minimum bactericidal concentration (mg/L); ^c^ by measuring the absorbance at 600 nm.

## Data Availability

The data used to support the findings of this study are included within the article, as well as the Supplementary Information File.

## Supplementary Materials

The following supporting information can be downloaded at: https://www.mdpi.com/article/10.3390/antibiotics13070609/s1, Figure S1: ^1^H-NMR of CAPP_1_ in CDCl3; Figure S2: ^1^H-NMR of CAPP2 in CDCl_3_; Figure S3: ^1^H-NMR of CAPP3 in CDCl_3_; Figure S4: ^1^H-NMR of CAPP4 in CDCl_3_; Figure S5: ^1^H-NMR of CAPP5 in CDCl_3._
