# Supplementary material for: A New Guanidine-Core Small-Molecule Compound as a Potential Antimicrobial Agent against Resistant Bacterial Strains
Source: Antibiotics (Basel). 2024 Jun 29;13(7):609. doi: 10.3390/antibiotics13070609 (PMC11274109; doi:10.3390/antibiotics13070609)
Supplement: Supplementary file 1 [file antibiotics-13-00609-s001.zip › antibiotics-3059006-supplementary.pdf]

A New Guanidine-Core Small-Molecule Compound as a Potential Antimicrobial Agent against Resistant Bacterial Strains

Figure S1. ^{1}H-NMR of CAPP1 in $CDCl_3$.

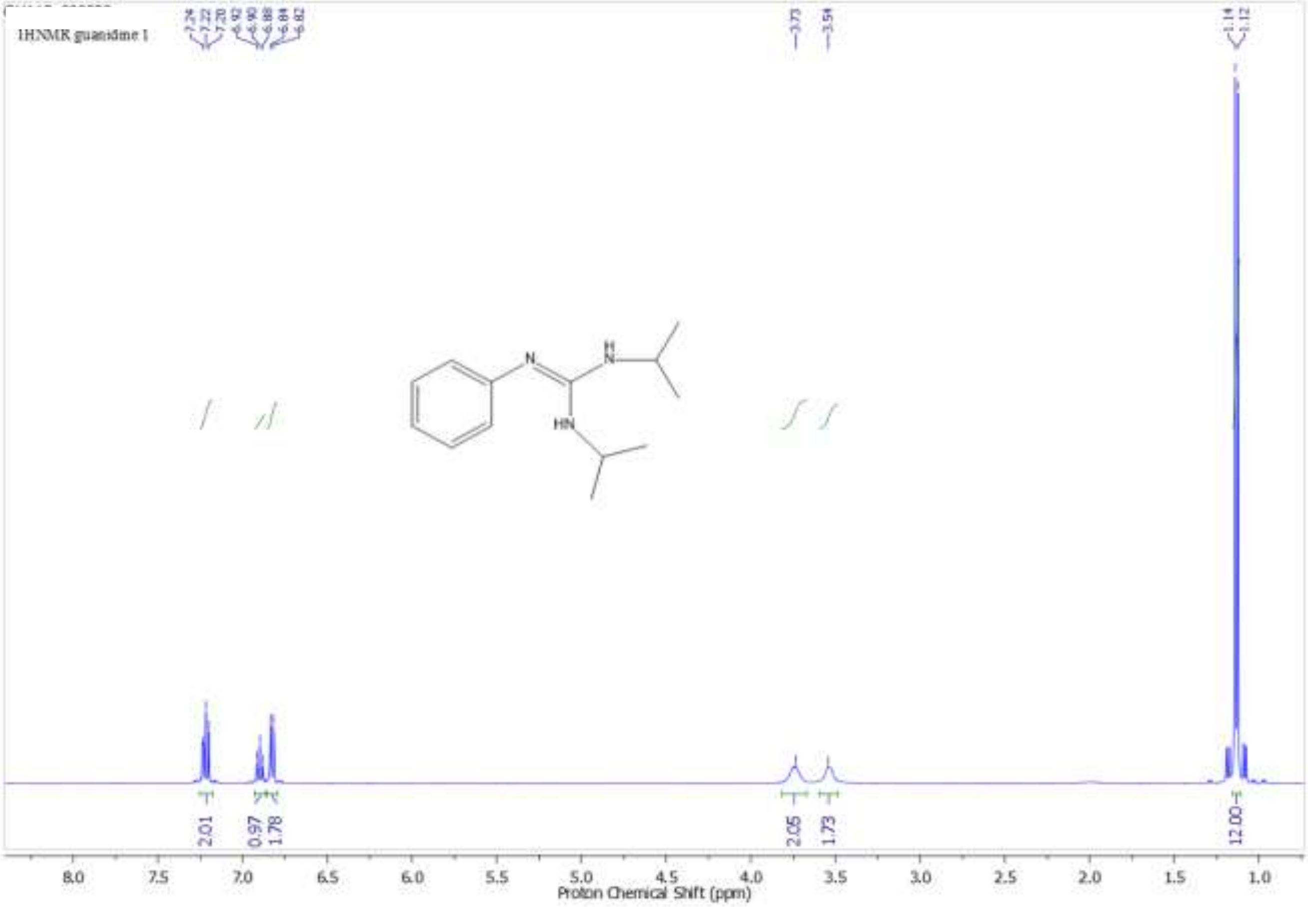

^{1}H NMR (400 MHz, $CDCl_3$): δ 7.22 (t, J = 6.9 Hz, 2H, CH-m-Ar), 6.90 (t, J = 7.4 Hz, 1H, CH-p-Ar), 6.83 (d, J = 8.4 Hz, 2H, CH-o-Ar), 3.74 (m, 2H, CH-isopropyl), 3.53 (bs, 2H, NH), 1.13 (d, J = 6.4 Hz, 12H, CH_3-isopropyl).

Figure S2. ^{1}H-NMR of CAPP2 in $CDCl_3$.

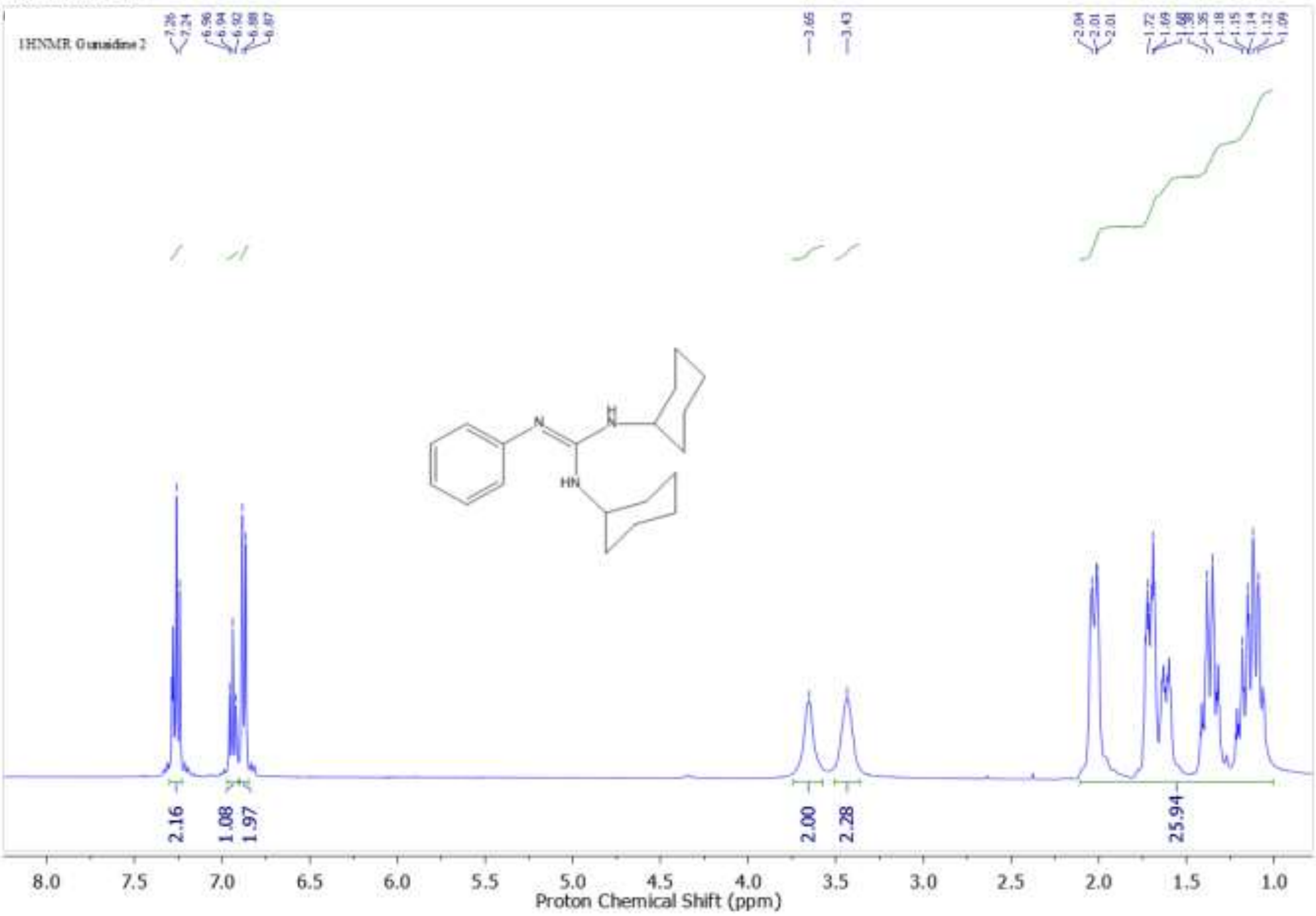

1H, CH-p-Ar), 6.88 (d, J = 7.3 Hz, 2H, CH-o-Ar), 3.65 (bs, 2H, NH), 3.43 (bs, 2H, CH-cyclohexyl), 2.11 – 1.02 (m, 20H, CH_2-cyclohexyl).

Figure S3. ^{1}H-NMR of CAPP3 in $CDCl_3$.

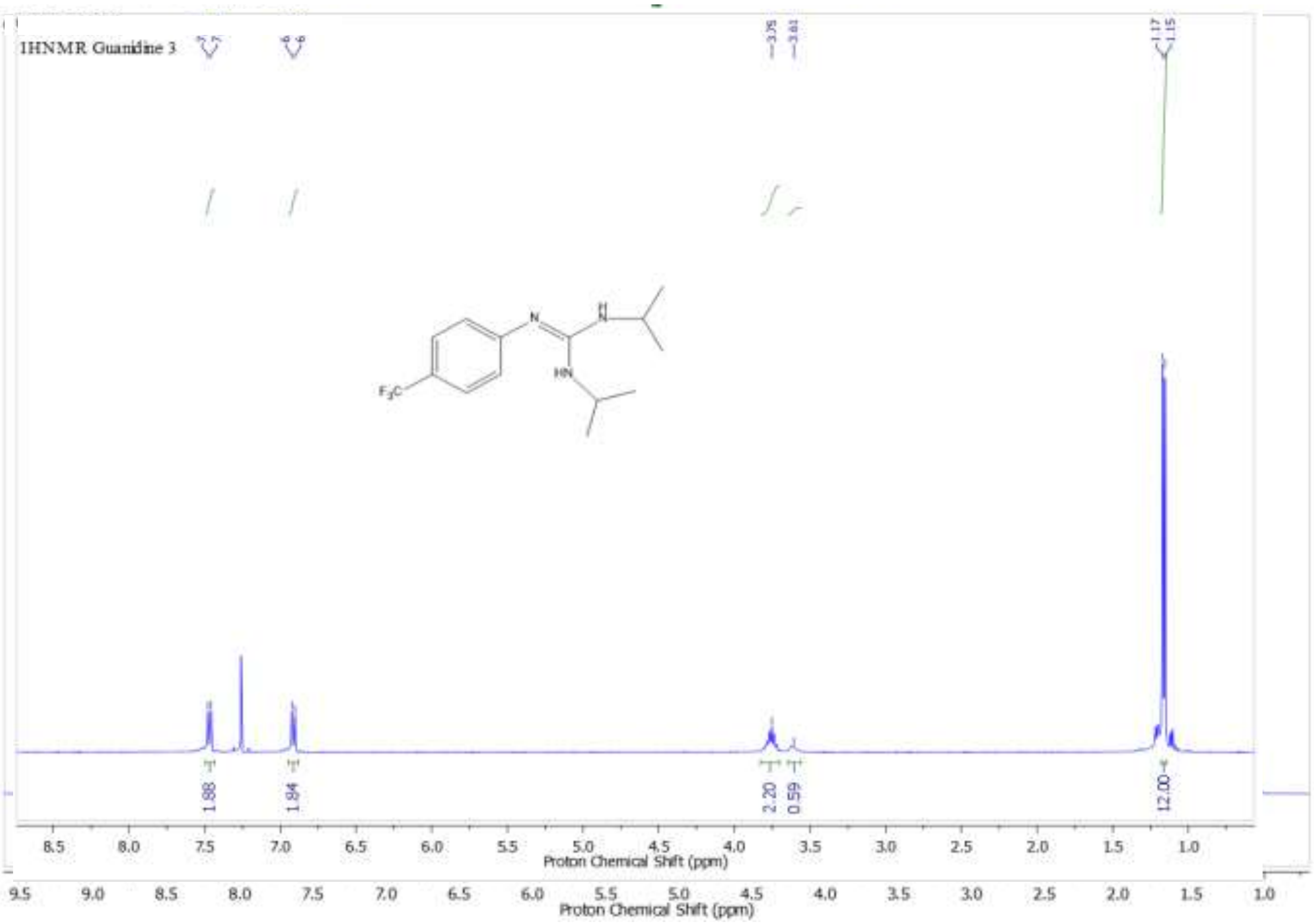

^{1}H NMR (400MHz, $CDCl_3$): δ 7.47 (d, J = 8.3 Hz, 2H, CH-o-Ar), 6.91 (d, J = 8.3 Hz, 2H, CH-m-Ar), 3.75 (m, 2H, CH-isopropyl), 3.62 (bs, 2H, NH), 1.16 (d, J = 6.4 Hz, 12H, CH_3-isopropyl).

Figure S4. ^{1}H-NMR of CAPP4 in $CDCl_3$

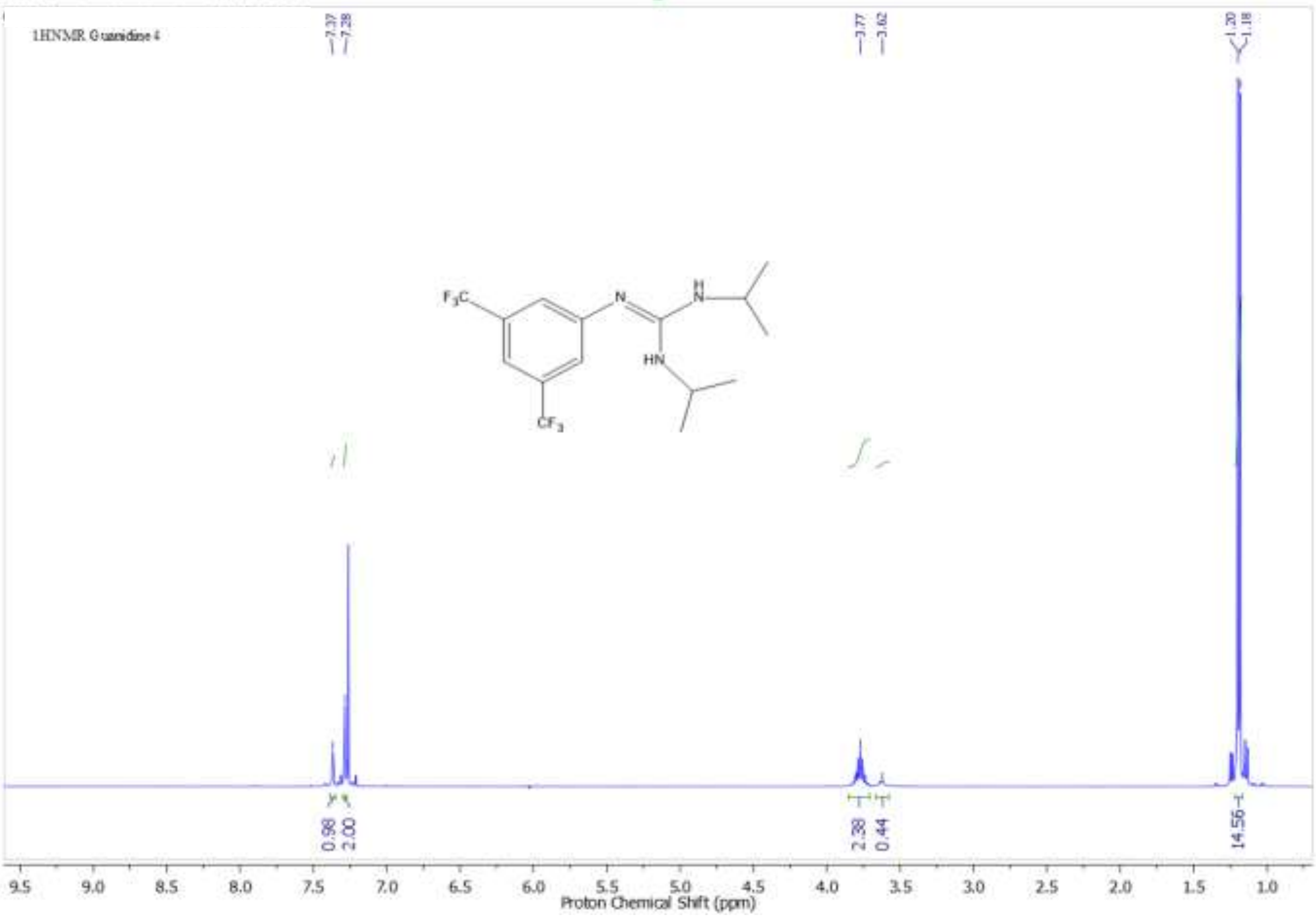

^{1}H NMR (400MHz, $CDCl_3$): δ 7.37 (s, 1H, CH-p-Ar), 7.28 (s, 2H, CH-o-Ar), 3.77 (m, 2H, CH-isopropyl), 3.62 (bs, 2H, NH), 1.19 (d, J = 6.4 Hz, 12H, CH_3-isopropyl).

Figure S5. ^{1}H-NMR of CAPP5 in $CDCl_3$

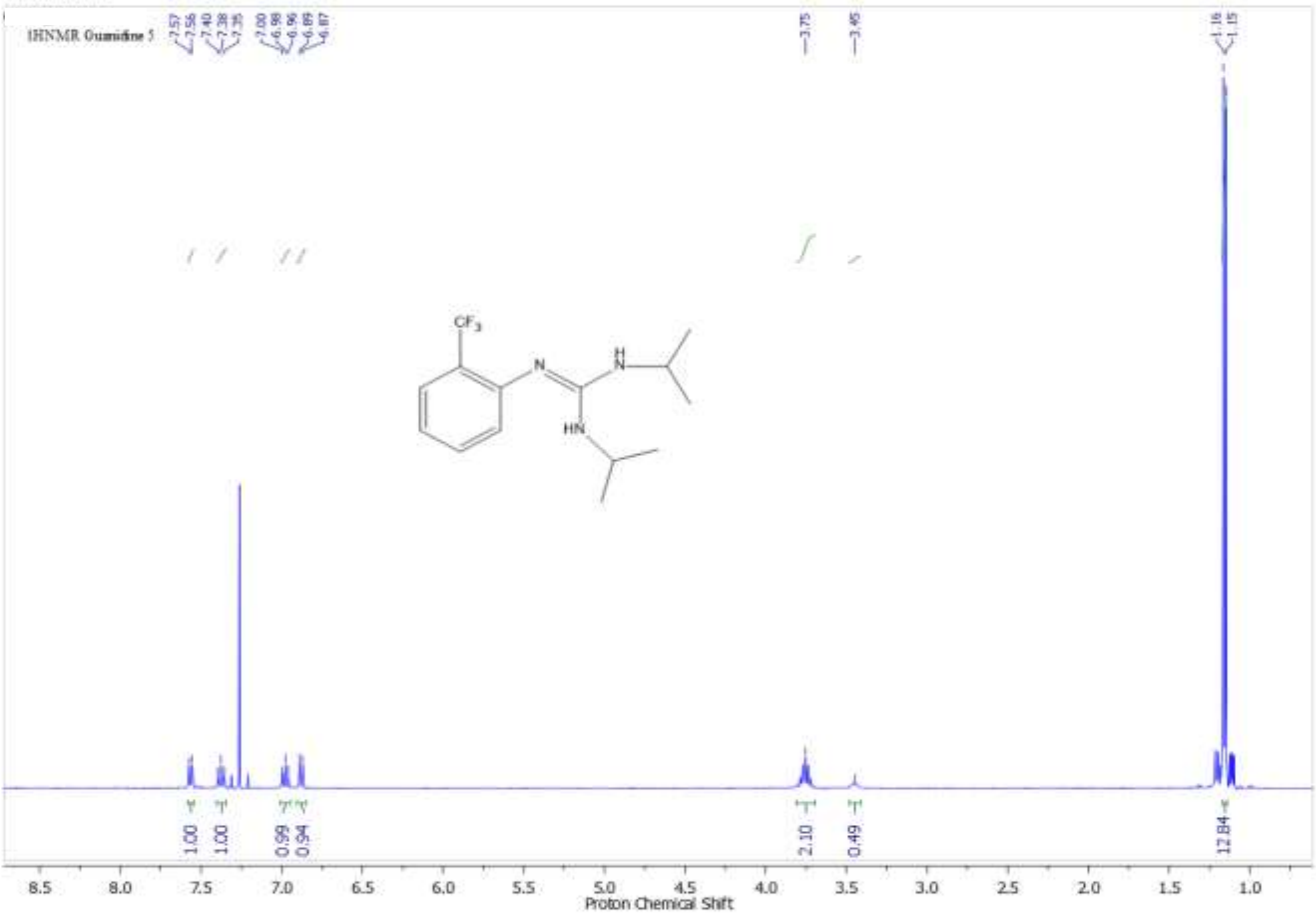

^{1}H NMR (400MHz, $CDCl_3$): δ 7.57 (d, J = 7.8 Hz, 1H, CH-Ar), 7.38 (t, J = 7.7 Hz, 1H, CH-Ar), 6.98 (t, J = 7.7 Hz, 1H, CH-Ar), 6.88 (d, J = 8.0 Hz, 1H, CH-Ar), 3.75 (m, 2H, CH-isopropyl), 3.45 (bs, 2H, NH), 1.15 (d, J = 6.4 Hz, 12H, CH_3-isopropyl).
